# Evaluation of a multimodal intervention to promote rational antibiotic use in primary care

**DOI:** 10.1186/s13756-021-00908-9

**Published:** 2021-04-06

**Authors:** Inga Petruschke, Florian Salm, Michelle Kaufmann, Antje Freytag, Jochen Gensichen, Michael Behnke, Tobias Siegfried Kramer, Regina Hanke, Petra Gastmeier, Sandra Schneider, RAI Study Group

**Affiliations:** 1 Institute of General Practice and Family Medicine, Faculty of Medicine, Friedrich-Schiller-University, Bachstrasse 18, 07743 Jena, Germany; 2 Institute for General Practice and Family Medicine, Ludwig-Maximilians-University/University Hospital, Pettenkoferstrasse 8/10, 80336 Munich, Germany; 3 Lindgrün GmbH, Cuxhavener Strasse 12, 10555 Berlin, Germany; 4 Charité – Universitätsmedizin Berlin, corporate member of Freie Universität Berlin and Humboldt-Universität zu Berlin, Institute of Hygiene and Environmental Medicine, Hindenburgdamm 27, 12203 Berlin, Germany

**Keywords:** Rational antibiotic use, Antimicrobial use, Acute respiratory tract infection, General practitioners, Outpatient care, Primary care

## Abstract

**Background:**

Increasing antimicrobial resistance is a serious societal challenge affecting outpatient, inpatient and veterinary care. The German *One-Health* project, RAI (Rational use of Antibiotics via Information and Communication) addresses all three sectors. In the outpatient sector, General Practitioners (GPs) are the main prescribers of antibiotics and were therefore, targeted for this study. A multimodal intervention focusing on Acute Respiratory Tract infections (ARI) was designed and implemented. The aim of this study was to evaluate acceptance, rating and the self-reported impact of the intervention among GPs.

**Methods:**

The intervention offered six tools: a GP training on rational antibiotic use, an app for self-monitoring, a leaflet and a set of posters (both for use as information materials in waiting rooms) and both digital and printed information prescriptions (material for ‘prescribing’ information instead of an antibiotic to the patient). The tools could be used according to individual preferences. The intervention was conducted between August 2016 and July 2017. Following the intervention, a three pages anonymous questionnaire was sent to all 271 participants. Items covered socio-demographic and professional background, use and judgement of the intervention tools (6 point Likert scale), impact of the intervention tools (4 point Likert scale).

**Results:**

The response rate was 39% (n = 107). On average, respondents used 3.1 of the six available tools, with printed information prescriptions used most frequently (79%). Digital information prescriptions were used more frequently by men than by women (OR 2.8; 95% CI 1.16–7.24; *p* = 0.02). Eighty-seven percent of respondents stated that information prescriptions supported doctor-patient communication. In a comparison of the overall impression of the different intervention tools the GP training on rational antibiotic use was rated best (1.67 on a 6 point scale with 1 = highest, 6 = lowest) and most often noted as having had a “strong” or “very strong” impact on personal antibiotic prescribing behavior.

**Conclusions:**

The multimodal intervention addressing education and communication was well accepted among GPs and could help in fostering rational use of antibiotics in primary care.

**Supplementary Information:**

The online version contains supplementary material available at 10.1186/s13756-021-00908-9.

## Introduction

In times of rising antimicrobial resistance, the prudent use of existing antibiotic substances and the avoidance of unnecessary prescriptions are essential [1]. Therefore, the study group of the German *One-Health* project RAI (Rational use of Antibiotics via Information and Communication) developed tailored interventions for veterinary, hospital and outpatient care. In the latter, General Practitioners (GPs) are the main prescribers [2]. It is likely that many antibiotics are still prescribed unnecessarily [3–5]. Acute Respiratory Tract infections (ARI) are among the most frequent infections in outpatient care [6] and are often treated with antibiotics [7, 8] despite the fact that the majority of ARIs are caused by viruses and/or are self-limiting [9]. Known barriers to appropriate use of antibiotics in the outpatient setting are: patients´ knowledge [10–12], patients´ expectations [13–15] and physicians’ characteristics [15–17]. When the study was planned, Germany ranked eighth in an EU comparison of systemic antibiotic use in outpatient care [18].

Several intervention strategies in primary care have proven to be effective in reducing unnecessary antibiotic prescriptions in ARI: doctor-patient communication [19–21], patient information leaflets [22], delayed prescribing [23, 24] and feedback to GPs [25–27]. Advanced training on rational antibiotic use is an important information source for German GPs and there is a corresponding demand for more training sessions that are independent of the pharmaceutical industry [28].

We therefore designed a multimodal intervention for GPs, which addresses the key aspects of physician and patient information, doctor-patient communication, and feedback of AB prescribing. Participants were offered a toolbox of knowledge and communication elements on the prudent use of antibiotics in ARI. The tools could be freely combined and complemented each other in terms of content.

The aim of this study was to describe acceptance, rating and the self-reported impact of the six intervention tools for GPs in order to understand what really works in everyday practice.

## Methods

### Design and setting

The multimodal intervention described here was embedded in a broader One-Health project called RAI: www.rai-projekt.de/rai/startseite/ [29]. In addition to GPs, the project targeted hospitals and livestock farming with different, tailored, interventions [30]. The project started in 2015, in three federal German states: Berlin, Brandenburg, Thuringia [28, 29, 31]. The intervention in primary care was tailored using information from interviews and workshops with GPs, a survey of GPs [15] and a survey of the German public [10]. All tools were developed in collaboration with professional communication designers specializing in human-centered design.

### Intervention

**Fig. 1:**
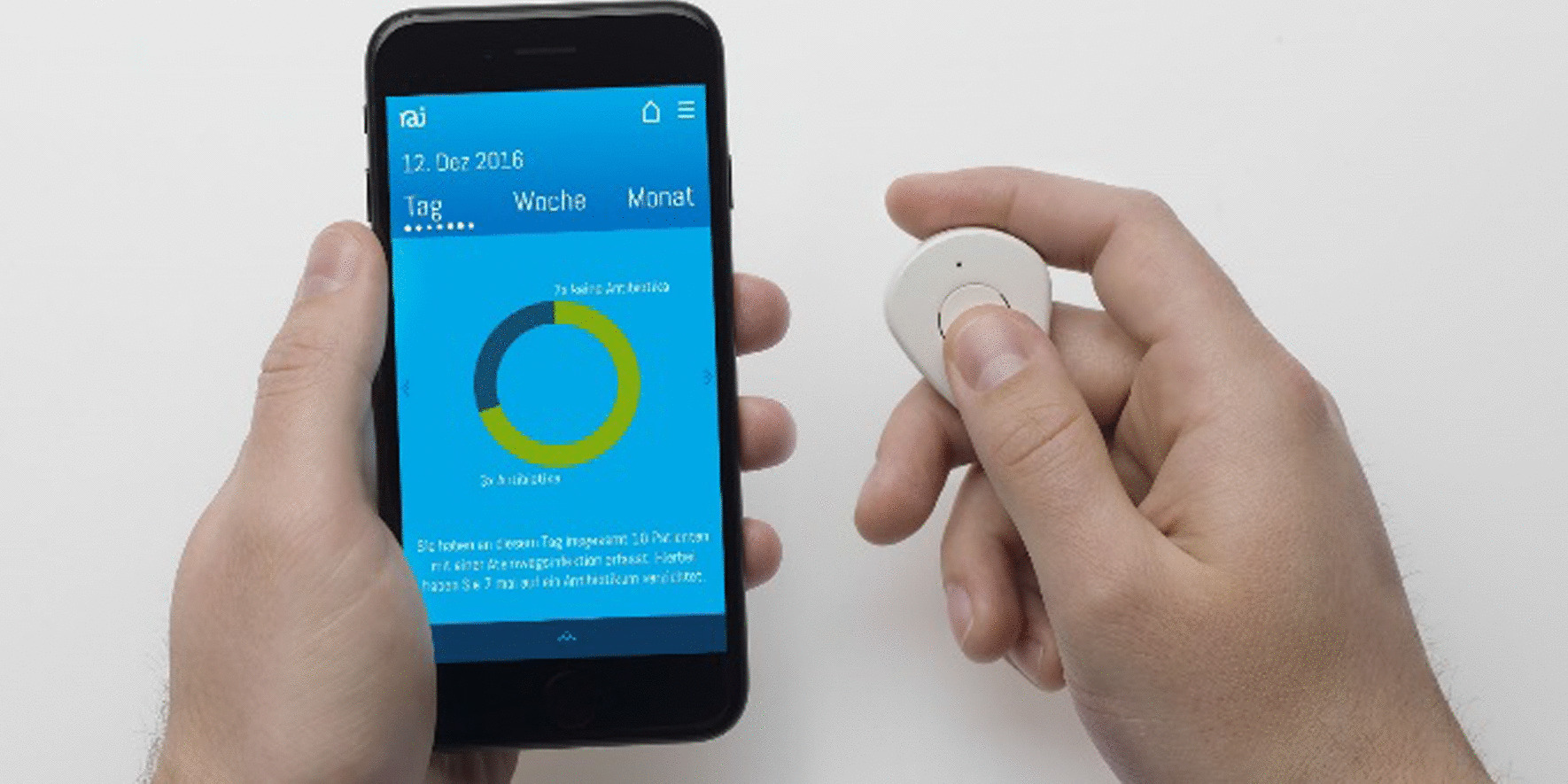
GP self-monitoring App. The user clicks the button (right) for every ARI-patient with (blue) or without (green) antibiotic treatment

**Fig. 2:**
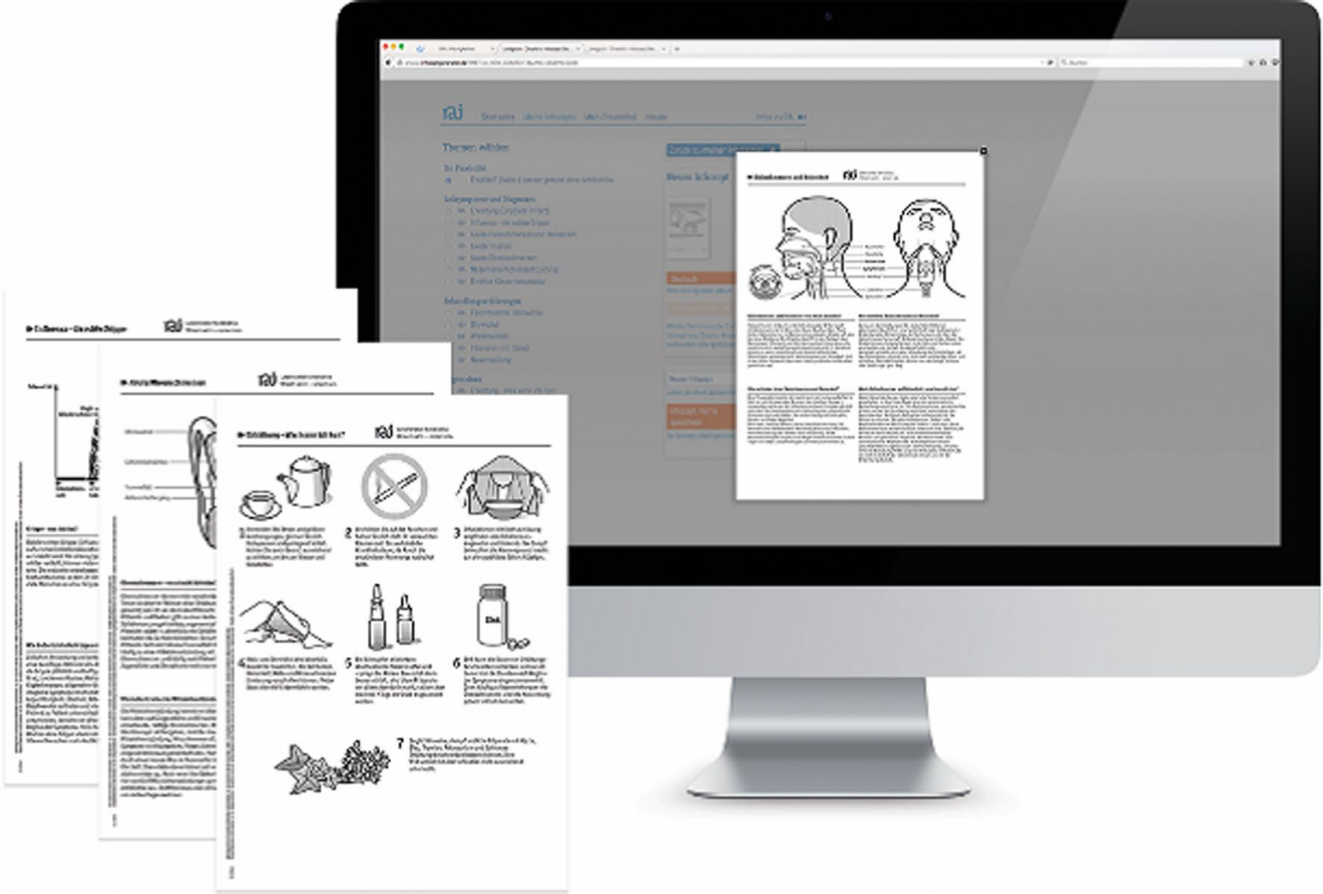
Digital information prescription

**Fig. 3:**
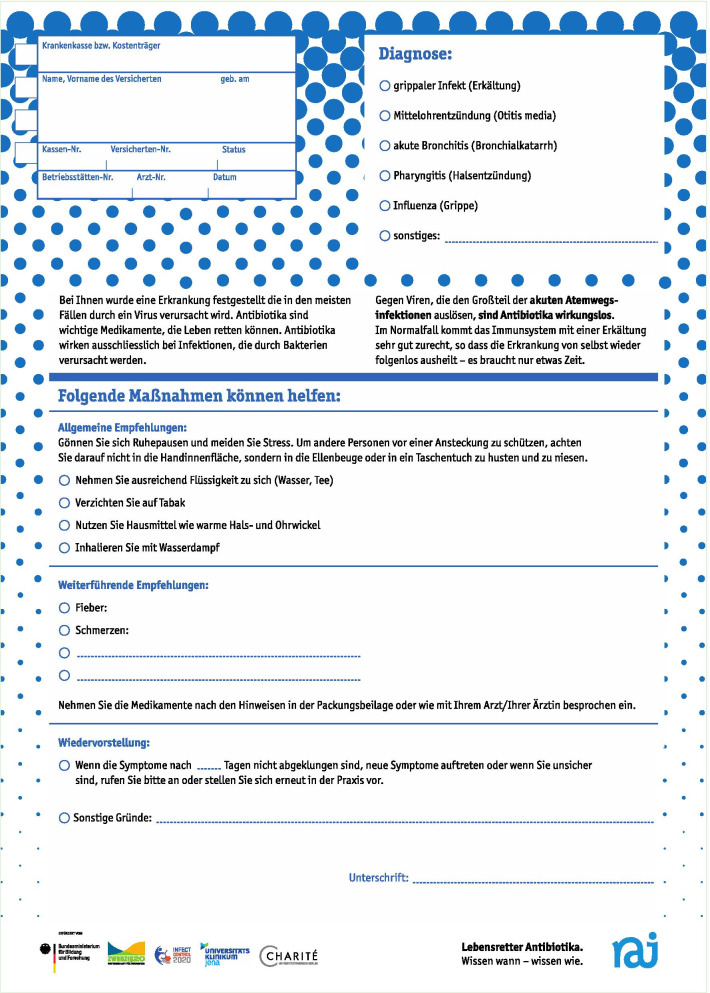
Printed information prescription

The primary care intervention aimed to address prescriber knowledge, self-monitoring of antibiotic prescriptions, patient information and support of doctor-patient communication. The following six intervention tools were developed:

1. GP training:
  A 2-h training session on rational antibiotic use, including:
  Development and epidemiology of antimicrobial resistance in Germany;
  Antibiotic use in outpatient care in Germany and Europe;
  Recommendations of antibiotic therapy in primary care (focused on ARI);
  Strategies to avoid unnecessary antibiotic prescriptions (including communication and how to apply RAI patient information).
  The training was free of charge and conducted 19 times in different locations in Berlin, Brandenburg and Thuringia between August 2016 and May 2017. It was certified by the German Medical Chamber. This was key as all medical specialists in Germany, including GPs, are required to complete a certain number of certified medical training hours in order to maintain their consultant status. 305 physicians took part in total. In the three federal states altogether 5.861 GPs are registered who would have been eligible for the training.
2. GP self-monitoring:
  A smartphone application for iOS^®^ was provided in which GPs could record the proportion of antibiotic prescriptions for ARI patients over time (daily profile, weekly profile, monthly profile or self-defined time window) by means of a brief single or double tap during consultations (Fig. 1).
3. Digital information prescriptions:
  Instead of prescribing antibiotics, material for ‘prescribing’ information to the patient was developed, called ‘information prescription’ (in German ‘Infozept’).
  15 different information prescriptions (IP, see Fig. 2) giving information regarding ARI symptoms and treatment options, including illustrated handling instructions for inhalation etc. were provided on an online platform. After login, participating GPs could individually compile selected information prescriptions for their patients and personalise printouts with their own office headings. Information prescriptions were available in German, English, Turkish and Arabic and were intended as handouts during consultation (see Additional file 1).
4. Printed information prescriptions:
  Paper information about symptoms and treatment of ARI were combined, with space for individual instructions for patients. Resembling a drugs prescription, the patient’s personal data (health insurance, date of birth and address), as well as the physician’s signature, could be added (Fig. 3). One-sided (DIN A4) and four-sided (DIN A5) versions were available.
5. Posters:
  Seven motives with messages in the above-named languages were provided. The theme was de-dramatising the common cold and underlining the lifesaving role of antibiotics in severe disease (DIN A0).
6. Waiting room patient leaflet:
  A waiting room information sheet was designed to be handed out to patients with suspected ARI by the receptionist. It contained information on different manifestations of ARI and the general course without antibiotics. The document contained a free space for the patient to write down questions for the doctor's consultation.

All tools were free of charge and could be used independently of each other. There was no required number or frequency of tools to be used; rather, the aim was to provide a choice of intervention tools.

### Recruitment

Participants were recruited via the above-described GP training sessions, announcements in regularly published online and print media, local and regional GP networks and the project website [31].

### Questionnaire

An anonymous, 28-question survey was developed, with print and online versions (LimeSurvey^®^ software). The questions addressed four topics:

- Socio-demographic and professional characteristics (8 items);
- Awareness of antimicrobial resistance (6 items);
- Use and rating of intervention tools (11 items), and;
- Impact of the tools on personal antibiotic prescribing behaviour (3 items).

The questionnaire also provided free text boxes to elaborate on why intervention tools were not used.

The paper questionnaire was sent by post to all participants of the study in July 2017. Additionally, the weblink was emailed to all participants with existing email addresses. The feedback period was five weeks. For item list, see Additional file 2.

### Statistical analysis

Printed replies were manually added to the online dataset. The dataset was checked for mistakes and double entries.

All analyses were performed using SPSS [IBM SPSS statistics, Somer, NY, USA].

Group differences were tested via chi-squared test. A *p*-value of < 0.05 was defined as significant.

Multivariable analysis was performed using linear, logistic or negative binomial regression models. Gender, age, type of practice (individual practice or group practice) and number of inhabitants of the place where the practice was located (as a measure of an urban or rural environment) were selected as independent variables.

## Results

### Participants

The GP training was open to the professional public and had no conditions attached. Only training attendees who either registered for study participation on site of the training or requested further materials afterwards were counted as study participants. Additionally, material requesters who had not attended training were also counted as study participants, as there were other recruitment channels besides the trainings. Altogether, we counted 271 GPs as study participants, of which 107 GPs returned the questionnaire (via post or online at 69 per cent and 31 per cent, respectively), resulting in a response rate of 39%. Nine respondents with a specialisation other than General Medicine were excluded from analysis, resulting in a sample of n = 107.

### Socio-demographic and professional characteristics

Socio-demographic and professional characteristics of the respondents are shown in Table 1. 57% of the respondents were female, the mean age was 51 years, and respondents had been working as GPs for an average of 13 years (range 0.5–33 years).

**Table 1:** Demographic and professional characteristics of the respondents

Parameter	
Female gender, n (%)	61 (57)
Mean age in years (SD; range)	50.8 (7.6; 32–80)
Mean professional experience in years (SD; range)	13.4 (8.4; 0.5–33)
Inhabitants at practice location, n (%)^a^	
< 5000	20 (18.7)
5000–19,000	29 (27.1)
20,000–99,000	17 (15.9)
> 100,000	38 (35.5)
Type of practice, n (%)^b^	
Single practice	57 (53.3)
Joint practice	41 (38.3)
Medical Care Centre (“MVZ”)	6 (5.6)

^a^3 missing data

^b^3 missing data

^c^1 missing data

### Use and rating of the intervention tools

**Fig. 4:**
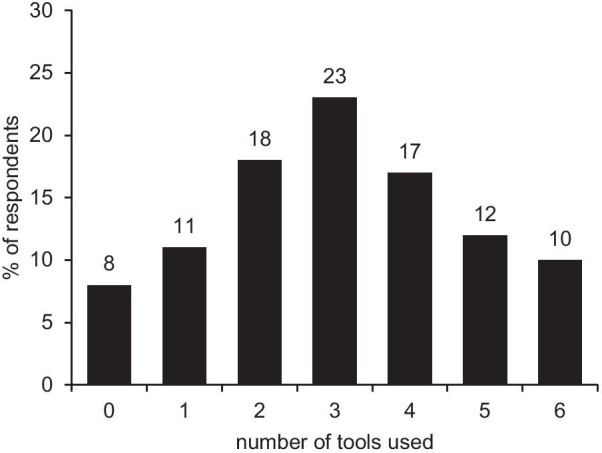
Number of tools used by the respondents (n = 107)

Respondents used an average of 3.1 of the six tools available. The distribution of how many respondents used how many tools is shown in Fig. 4.

Negative binomial regression did not show any significant association between socio-demographic characteristics and the number of tools used.

The most frequently used tool was the printed IP (85 respondents/79%). Approximately half of these also used digital IP (43 respondents/40%), with one respondent using digital IP exclusively. 64% of the respondents used the waiting room patient leaflet and 55% used posters. Half of the respondents (n = 55/51%) attended a training session. The least-used tool was the GP self-monitoring App for iOS^®^ (16%)—see Table 2. The most frequently stated reason for not having used the app was a lack of technical prerequisites (28/59), e.g. *“I do not own a smartphone”.*

**Table 2:** use and rating of the different intervention tools

Tool	Users n (%)^a^	Average rating six-point scale (SD)
GP training on rational antibiotic use	55 (51.4)	1.67 (1.171)
GP self-monitoring app	17 (15.9)	1.76 (0.903)
Printed information prescription	85 (79.4)	1.79 (1.100)
Digital information prescription	44 (41.1)	1.80 (1.091)
Posters	59 (55.1)	2.05 (1.166)
Waiting room patient leaflet	68 (63.6)	1.75 (0.983)

^a^Users are defined as respondents who rated the tools

The tools were rated on average at 1.79 on a six-point scale, where 1 = highest and 6 = lowest. The questionnaire asked for an “overall rating” which includes aspects like usability and perceived effectiveness. GP training on rational antibiotic use was the highest rated, with a mean of 1.67. Posters were the lowest rated, with a mean of 2.05.

### Digital versus printed information prescriptions

**Fig. 5:**
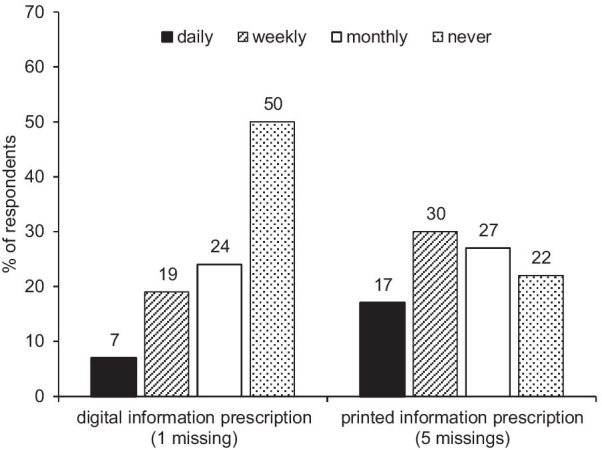
Frequency of use of digital or printed IPs in everyday work

Participants were asked how often they used the information prescriptions in their everyday work: compared to digital IP, the printed IP were used more frequently. 47% of respondents used them at least weekly. Digital IP were used at least weekly by 26% of respondents—see Fig. 5.

In the free text field, respondents gave additional information on why they did not use the tools:

The most frequently stated reason for not using the digital IP was missing technical prerequisites (n = 16/48), e.g. *“I do not have internet in the consultation room”.*

For respondents who did not use the printed IP, the most common reason was no necessity for it (n = 7/21), e.g. *“Patients want consolation rather than information”*.

Digital IP were described as supportive in doctor-patient-communication by 39 respondents (36%) and printed IP by 69 respondents (64%).

Multivariable logistic regression analysis, including socio-demographic characteristics, showed that digital IP were used more frequently by men than by women (OR 2.9; 95% CI 1.155–7.238; *p* = 0.023). This result was not found for printed IP.

The translation feature of the digital IP was of use to 22 of the respondents (21%). Seven respondents (7%) stated that the digital IP were missing a language they required. Four respondents (4%) suggested adding patient information on acute cystitis. 16 (15%) of the respondents wished to individualise the digital IP by adding the name of the patient. 31 of the respondents (29%) wished to integrate the digital IP into their medical software programme.

### Influence on antibiotic prescribing behaviour

GPs judged the intervention tools differently regarding influence on personal antibiotic prescribing behaviour—see Table 3.

**Table 3:** Answers to the question “How would you rate the influence of the different tools on your antibiotic prescribing behaviour?” (4-point scale)

Intervention tools	“Strong” or “very strong” n (%)
GP training on rational antibiotic use	56 (52.3)
Self-monitoring App	12 (11.2)
Information prescription (digital and printed)	47 (43.9)
Waiting room patient information (poster and leaflet)	39 (36.4)
Participation in the study	55 (51.4)

The highest proportion of respondents (52%) found that the GP training on rational antibiotic use had a strong/very strong influence on their personal antibiotic prescribing behaviour, followed by participation in the study (51%). This was confirmed by the frequency of participants noting, within the free text quote section, that participation in the study had encouraged their existing prescribing behaviour, e.g., *“I feel confirmed in my prescribing behaviour*”. Still, relevant proportions of respondents confirmed a strong influence on their prescribing behaviour for IP (44%) and for the waiting room patient information (36%).

Multivariable logistic regression analysis revealed that self-reported influence of GP training on rational antibiotic use was stronger in men than in women (OR 5.3; 95% CI 1.560–18.297; *p* = 0.008). Influence of participation in the study on antibiotic prescribing behaviour was stronger in GPs working in a joint practice than in GPs working in a single practice (OR 3.3; 95%CI 1.151–9.495, *p* = 0.026). Influence of waiting room patient information on individual prescribing behaviour was stronger in men (OR 3.3, 95%CI 1.331–8.382, *p* = 0.01).

## Discussion

In our study, we evaluated a tailored multimodal intervention for GPs, developed with human-centered design methods.

The response rate to our questionnaire was 39%, which is comparable to other surveys in primary care [15, 32].

Overall, the intervention tools were well accepted and highly rated. Most respondents used more than one tool, with an average use of 3.1 of the six available intervention tools. A choice of different tools increases the likelihood of meeting different physician’s preferences.

The GP training on rational antibiotic use was the best-rated tool, with a “strong” or “very strong” influence on antibiotic prescribing behaviour by all training session attendees. This emphasizes the importance of training on the topic for GPs and is in line with our survey from the first project phase, where we explored the information sources of our target groups [28]. GP education has proven effective in the reduction of inappropriate antibiotic prescribing in primary care [27] and has been part of multifaceted interventions [33]. However, training on rational antibiotic use without pharmaceutical sponsoring are scarce in the outpatient sector in Germany. Our findings confirm that training on rational antibiotic use should firmly be integrated into GPs’ continuing medical education. To extend coverage, we developed online training (MOOC), which was used by a significant number of physicians after the intervention period.

Despite its good rating, GP training was not the most frequently used tool in our study. Approximately half of the respondents stated that they participated in one of the training sessions. Overall, over 300 physicians took part in our GP training. However, only those who later ordered additional material (i.e. tools) were registered as study participants. This is due to the certification procedure of the Medical Chamber for elective courses. Certified courses may not be part of a study.

On the other hand, the fact that nearly half of the respondents did not participate in the training shows that, in addition to the public announcement of the training, our other recruitment measures were also effective. Fewer personnel-consuming intervention tools could also be distributed without training as an anchor point.

At the time of the study, it was not possible to access prescriber feedback on antibiotic use from claims data in Germany. We therefore aimed to develop a simple application for GP self-monitoring. Compared to the other tools, the GP self-monitoring app was the least-used intervention tool. A frequently mentioned reason for that was a lack of technical prerequisites. Our budget only allowed for app development for one smartphone type; we chose the iOS^®^, but it is possible this was not best suited for the target group. Additionally, even if it was only one single tap (ARI without antibiotic prescription) or double tap (ARI with antibiotic prescription), it is an extra effort to actively use the application systemically, which might have overburdened already busy GPs. However, the small group who did use the app rated it as good; in cohorts with appropriate technical prerequisites and with higher digital affinity, the idea could work.

Most patients expect examination and explanation of their symptoms, rather than antibiotics [32, 34–36]. On the other hand, doctors often feel a certain AB prescription pressure exerted by the patient [19]. Fostering doctor-patient communication is an effective strategy to address this dilemma [37]. However, in Germany, a GP has an average of 8 min per patient [38] and, therefore, needs a time-effective tool. To address these needs, we developed information prescriptions, similar to ´viral prescription pads´ used in Northern America [39]. Internationally, such tools have already shown positive effects on the reduction of unnecessary antibiotics [22]. Furthermore, they correspond with the participatory approach in primary care, by increasing health competence of patients.

Printed information prescriptions were by far the most-used tool from our set. They were used twice as often as the digital version and more frequently in everyday work. Overall, half of those who used printed IPs also used digital versions, but only one person used digital IPs exclusively. Thus, in our responder cohort, digital IPs were a complement rather than a substitute for the printed versions. Printed IPs were short and ‘ready to use’, while the online platform provided a larger variety of content, with a broad choice of symptoms, handling instructions in several languages and the requirement of basic technical equipment (internet access), logging in, identification of relevant information for the patient, and printing. Hence, one could imagine that the printed versions were used preferentially for ‘standard’ patients and the digital content for patients that are more ‘complex’. However, what we can learn from this is that a considerable proportion of our cohort is not limited to digital or analogue procedures in their offices, but uses a combination of the two. Important for software developers is the finding that 31 of the 44 respondents who used the digital IP wanted it to be integrated with their medical software. Digital IPs were used more frequently by men than by women, which points towards different physician preferences and emphasizes the importance of diversity in intervention tools.

Overall, information prescriptions were used by 80% (n = 85) of respondents, of whom 40% (n = 43) used both types; this was possibly also due to different preferences of different patients.

The better-informed patients are, then the better they cooperate [40, 41]. Waiting room patient leaflet and posters were frequently used. By addressing patients before consultation, they served as preparation and, thereby, supported doctor-patient communication.

Apart from the GP self-monitoring app, all tools were well-accepted and respondents used them in a variety of combinations. Over half the respondents stated “*participation in the study”* itself had a “strong” or “very strong” influence on their individual prescribing behaviour. These findings suggest that the overall concept of a multimodal intervention (“tool box”), rather than a single tool, was convincing.

## Limitations

1. The sample of participants in the study, and hence the respondents, might be GPs who are already aware of the subject and make rational decisions about antibiotic use (self-selection bias).
2. Respondents might have given socially desirable answers.
3. The project was located in Eastern Germany, where there are lower antibiotic prescribing rates for systemic antibiotics in the outpatient setting, compared to Western Germany [42]. Therefore, the level of study participants’ room for improvement could be lower than in Western Germany.
4. As a subjective parameter, self-reported change of antibiotic prescribing behaviour is of limited value in describing the impact of intervention tools. Analysis of the effect on prescription data is pending.

## Conclusions

A tailored intervention with six different tools was developed and evaluated. The high acceptance and use of several, but rarely all, tools shows that a multimodal concept makes sense. Training and educational concepts should be further developed and disseminated. Waiting room information is a useful addition. Future app developments should be preceded by an analysis of the technical prerequisites of the target group.

In principle, analogue and digital measures can be applied in primary care interventions. At present, however, a combination of the two is advisable in Germany.

## Supplementary Information

**Additional file 1.** Digital information prescription in four languages.

**Additional file 2.** List of questionnaire items (translated from German).

## Data Availability

The datasets used and/or analysed during the current study are available from the corresponding author on reasonable request.

## Abbreviations

RAI: Rational use of antibiotics via information and communication
GP: General practitioner
ARI: Acute respiratory tract infection
IP: Information prescription
