# Supplementary material for: Evaluation of a multimodal intervention to promote rational antibiotic use in primary care
Source: Antimicrob Resist Infect Control. 2021 Apr 6;10:66. doi: 10.1186/s13756-021-00908-9 (PMC8025382; doi:10.1186/s13756-021-00908-9)
Supplement: Supplementary file 1 — Additional file 1. Digital information prescription in four languages. [file 13756_2021_908_MOESM1_ESM.pdf]

Akuter Husten

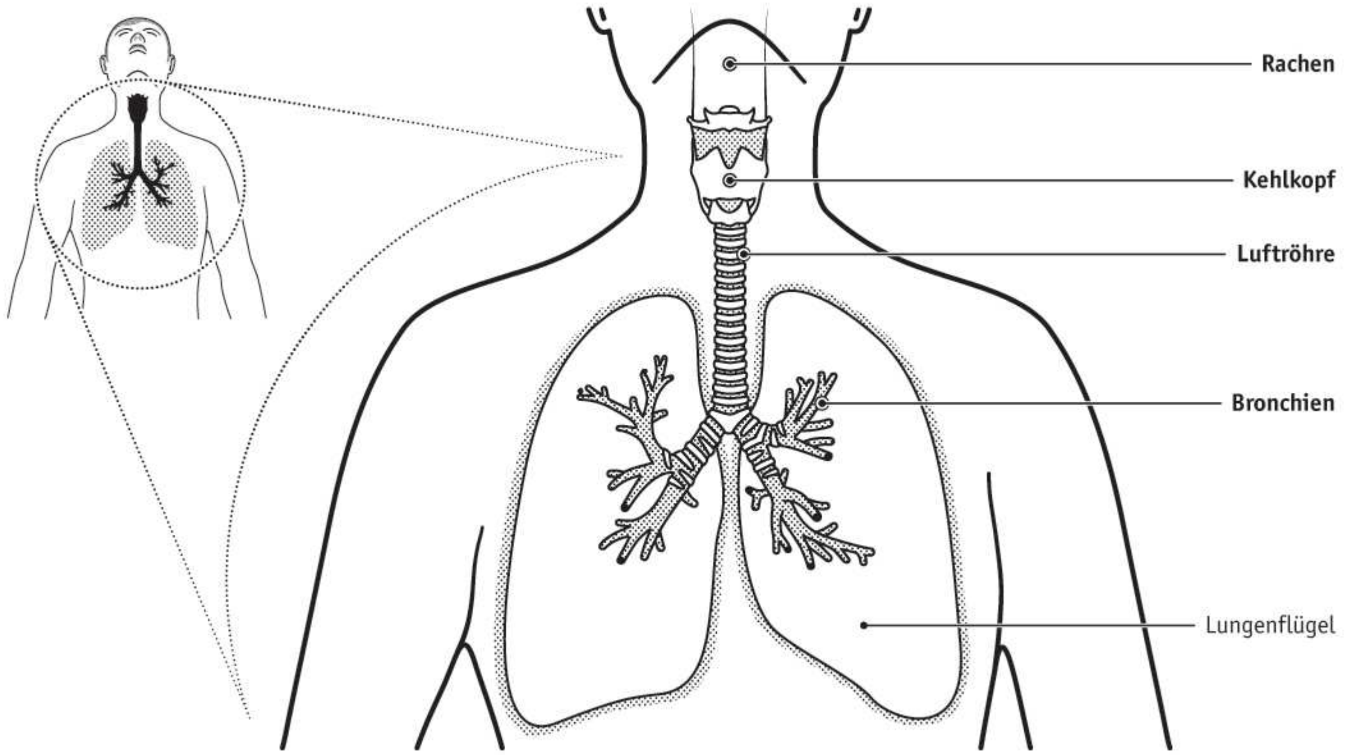

Husten — was steckt dahinter?

Husten ist ein wichtiger Schutzreflex des menschlichen Körpers. Um Schleim, Staub oder Fremdkörper aus den Atemwegen zu entfernen, wird dabei explosionsartig Luft ausgestoßen – mit der Geschwindigkeit eines Hurrikans. Verantwortlich dafür sind spezielle Rezeptoren, die sich in den Bronchien, der Luftröhre, dem Kehlkopf und dem Rachen befinden. Werden diese Sensoren gereizt, beispielsweise durch Schleimansammlungen, senden sie Signale an das Gehirn, das daraufhin den Hustenreflex auslöst. Neben dieser unwillkürlich ablaufenden Reaktion kann man auch bewusst und absichtlich husten.

Wie entsteht Husten?

Beim Husten handelt es sich nicht um eine eigenständige Erkrankung, sondern um ein Symptom, das bei verschiedenen Krankheitsbildern auftreten kann. Zu den häufigsten Auslösern gehören akute Atemwegsinfekte wie die Erkältung und die Bronchitis. Die zumeist durch Viren bedingte Infektion führt in den Atemwegen zu einer Entzündung und - damit einhergehend oder etwas verzögert – zu einer verstärkten Schleimproduktion. Beides bewirkt dann den Hustenreiz. Für das Sauberhalten der Bronchien sind eigentlich Millionen von Flimmerhärchen zuständig, die sich dort in der Schleimhaut finden. Bei einer Infektion kann dieses Reinigungssystem an seine Grenzen kommen. Dann hilft der Husten, den zähen Schleim und mit ihm die Krankheitserreger aus den Atemwegen abzutransportieren.

Wie äußert sich Husten?

Zu Beginn ist der Husten bei Atemwegsinfekten meist trocken. Wenig später macht sich dann die verstärkte Produktion von Schleim bemerkbar, der mit dem Hustenstoß in den Mundraum gelangt. Manchmal sieht der Schleim weißlich-klar aus, er kann aber ebenso gelblich oder grünlich verfärbt sein. Letzteres bedeutet jedoch nicht, dass es sich um eine bakterielle Infektion handeln muss.
Begleitet wird der Husten oft von weiteren Symptomen des Infekts wie Schnupfen, Halsschmerzen, Heiserkeit, Kopfschmerzen, Abgeschlagenheit und manchmal auch von Fieber. Diese Beschwerden klingen in der Regel binnen einiger Tage wieder ab. Der Husten hingegen kann nach einer Erkältung, einer Grippe oder einer Bronchitis noch bis zu vier Wochen anhalten.

Akuter Husten – was kann ich tun?

Atemwegsinfektionen und der damit einhergehende Husten heilen in der Regel auch ohne Medikamente folgenlos aus. Da es sich um einen wichtigen Reinigungsmechanismus handelt, sollten Hustenstiller nur in Ausnahmefällen eingesetzt werden. Auch schleimlösende Medikamente sind meist nicht notwendig. Inhalationen mit Wasserdampf empfinden viele Patienten als angenehm. Unterstützend können pflanzliche Präparate, zum Beispiel auf Thymian-Basis eingenommen werden, obwohl es zu deren Wirksamkeit nur wenige Studien gibt. Wichtig ist es genügend aber nicht übermäßig viel zu trinken. Körperliche Schonung hilft ebenfalls beim Gesundwerden. Zigarettenrauch – ob aktiv oder passiv – gilt es bei Husten zu meiden. Antibiotika, bei bakteriellen Infektionen oft lebensrettend, haben gegen Viren, die Atemwegsinfekte meist verursachen, keine Wirkung. Deshalb ist eine Antibiotikatherapie bei Husten im Normalfall nicht nötig. Ausnahme ist die bakterielle Lungenentzündung, die jedoch nur einen sehr kleinen Anteil aller Infektionen der Atemwege ausmacht.

Verantwortlich für diese Patienten-Information ist die Charité (Berlin) und die Friedrich-Schiller-Universität (Jena) im Rahmen des RAI-Projektes (www.rai-projekt.de). Copyright Lindgrün GmbH Berlin 2016.
Diese Patienten-Information ist für Sie, als Erwachsene/r, persönlich. Sie sollte weder zur Selbstdiagnose noch zur Therapie ohne ärztliche Unterstützung eingesetzt werden. Bei Fragen wenden Sie sich an Ihren behandelnden Arzt. Empfehlungen & Informationen können für Kinder unzutreffend sein.
9 / 2016

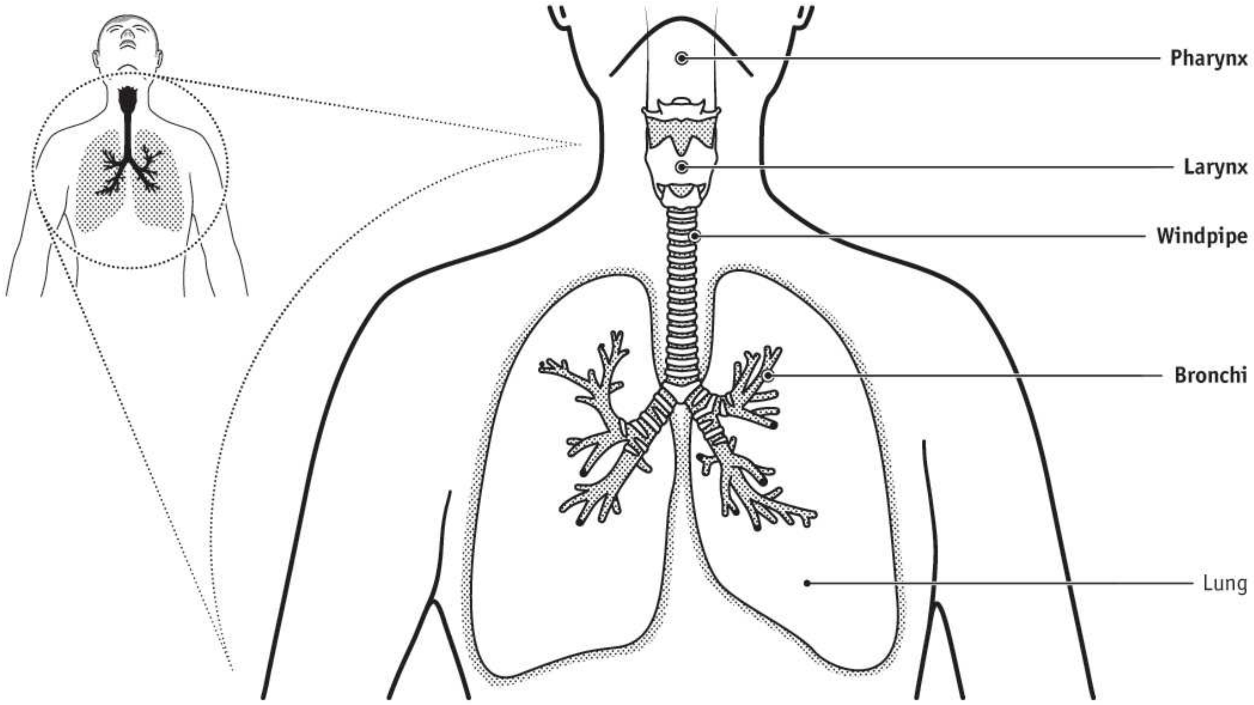

Coughing - What causes it?

Coughing is an important defensive reflex of the human body. In order to remove mucus, dust or foreign bodies from the respiratory tract, air is emitted like an explosion - with the speed of a hurricane. Special receptors are responsible for this occurrence, which are located in the bronchial tubes, windpipe, larynx and the pharynx. If these sensors are irritated e.g. by the accumulation of mucus, they send signals to the brain, which then triggers a cough reflex. Besides this spontaneous reaction, one can also cough consciously and intentionally.

How does coughing develop?

Coughing is not an independent disease, but it is a symptom that occurs in various diseases. Acute respiratory tract infections such as cold and bronchitis are the most significant trigger factors. Infections mostly caused by viruses lead to an inflammation in the respiratory tract and – along with it or soon after – to an increased mucus production. Both these occurrences cause an irritation of the throat. Millions of cilia existing in the mucous membrane are responsible for keeping the bronchi clean. In case of an infection, this cleaning system reaches its limits and the coughing helps in throwing out the viscous mucus and along with it, the pathogenic agent out of the respiratory tracts.

What are the symptoms of coughing?

Coughing in respiratory tract infections is mostly dry in the beginning. Subsequently, the increased production of mucus that enters into the mouth with the bout of coughing becomes more conspicuous. Sometimes the mucus is white-transparent, but it can also be yellowish or greenish in color. The latter does not mean that the person has a bacterial infection.
Coughing is often accompanied by other symptoms of the infection such as catarrh, sore throat, hoarseness, headache, fatigue and even fever. These symptoms usually subside within a few days. But the coughing can last up to four weeks following a cold, flu or bronchitis.

Acute coughing - What can I do?

Respiratory tract infections and coughing associated with them usually heal even without medicines and with no consequences. As this concerns an important cleaning mechanism, cough suppressants should be used only in exceptional cases. Many patients feel better with steam inhalation. Herbal supplements can be taken to support the healing process such as supplements based on thyme, although hardly any research has been carried out to examine their effectiveness. An intake of adequate but not excessive fluids is important. Avoiding physical exertion helps in the healing process. Smoking - active or passive - must be avoided if you are coughing. Antibiotics often act as lifesavers when you suffer from bacterial infection, however they do not have any effect against the majority of respiratory tract infections, which are usually caused by viruses. Therefore antibiotic treatment is usually not necessary for coughing. An exceptional case is the bacterial lung infection that constitutes a very small percentage of all the infections of the respiratory tract.

Charite (Berlin) and the Friedrich-Schiller-University (Jena) are responsibe for this patient information as a part of the RAI - Project (www.rai-projekt.de). Copyright Lindgrün GmbH Berlin 2016.
This patient information is personal for you as an adult. It should neither be used for self - diagnosis nor for treatment without medical support. In case of queries, please contact the doctor treating you. Recommendations and information are not applicable to children.

9 / 2016

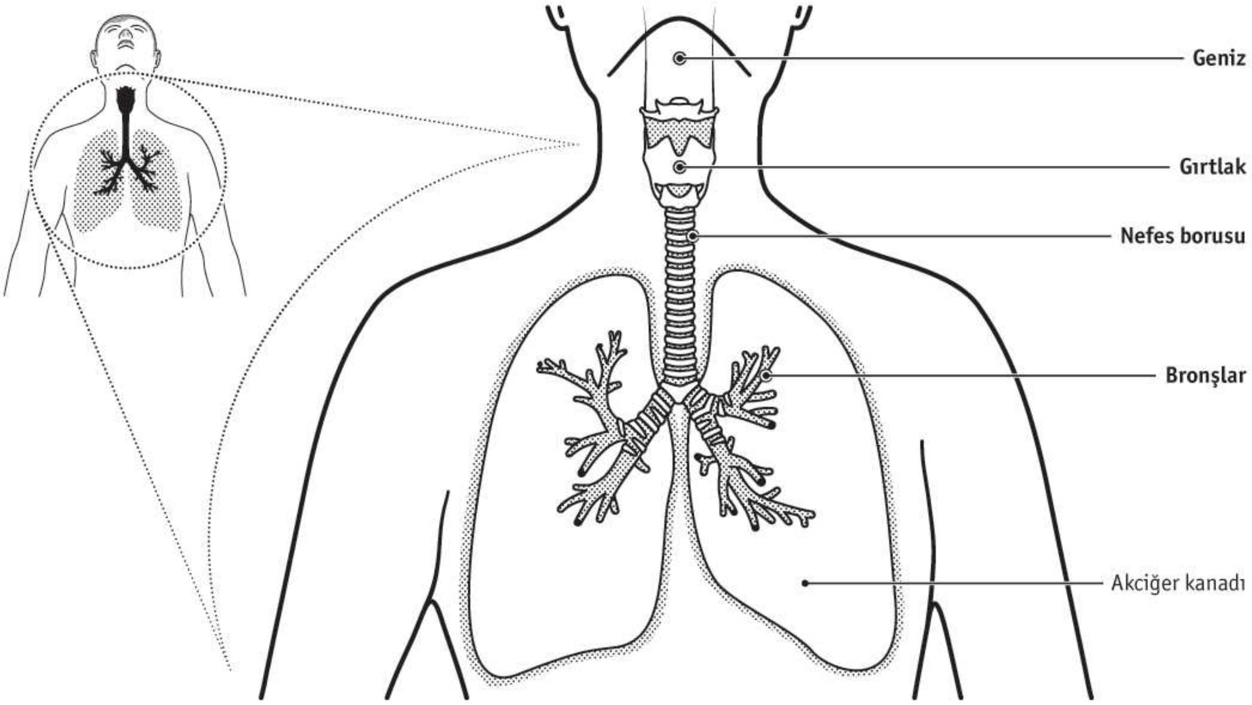

Oksürük nedir?

Oksürmek, insan vücudunun önemli bir koruma refleksidir. Balgamı, tozu veya yabancı cisimleri solunum yollarından uzaklaştırmak için patlama şeklinde ve bir fırtına hızında dışarı hava atılır. Bunun sorumlusu bronşlarda, nefes borusunda, gırtlakta ve genizde bulunan özel reseptörlerdir. Örneğin balgam birikmesi nedeniyle bu duyargalar tahrik olduğunda beyine sinyal iletilir ve bunun üzerine de öksürme refleksi tetiklenir. İstenmeden gerçekleşen bu refleksin yanı sıra bilinçli olarak ve bilerek de öksürmek mümkündür.

Oksürük nasıl oluşur?

Oksürük kendi başına bir hastalık değil, çeşitli hastalıklarda görülebilen bir semptomdur. Buna en sık neden olan hastalıklar ise soğuk algınlığı ve bronşit gibi akut solunum yolları enfeksiyonlarıdır. Genellikle virüslerin neden olduğu enfeksiyon, solunum yollarında iltihaplanmaya ve bununla birlikte ya da biraz gecikmeli olarak yoğun balgam üretimine neden olur. Her ikisi de öksürük gıcığını tetikler. Bronşların temiz kalmasından aslında buradaki mukozada bulunan milyonlarca tüy sorumludur. Bir enfeksiyon halinde bu temizlik sistemi sınırlarını zorlayabilir. Bu durumda öksürük, katı balgamı ve bununla birlikte hastalık virüslerini solunum yollarından dışarı atmaya yardımcı olur.

Oksürük kendini nasıl gösterir?

Solunum yolları enfeksiyonlarında öksürük ilk başta genellikle kurudur. Bir süre sonra öksürüğün neden olduğu darbeyle ağız bölgesine ulaşan balgam üretiminin arttığı gözlemlenir. Bazen balgam beyaz-şeffaftır, ancak bazen de sarımsı veya yeşilimsi olabilir. Ancak yeşilimsi oluşu illa bir bakteriyel enfeksiyonun söz konusu olduğu anlamına gelmez. Öksürüğe genellikle nezle, boğaz ağrısı, ses kısıklığı, baş ağrısı, bitkinlik ve bazen de ateş gibi enfeksiyonun diğer semptomları eşlik eder. Bu şikayetler genellikle birkaç gün içinde yok olur. Ancak öksürük, bir soğuk algınlığından, gripten veya bronşitten sonra dört hafta daha devam edebilir.

Akut öksürükte ne yapabilirim?

Solunum yolu enfeksiyonları ve beraberinde getirdikleri öksürük genellikle ilaç kullanılmadan problemsizce iyileşir. Söz konusu önemli bir temizleme mekanizması olduğundan öksürüğü dindiren ilaçlar sadece istisnai durumlarda kullanılmalıdır. Balgam sökücü ilaçlara da genellikle ihtiyaç yoktur. Birçok hasta su buharı inhalasyonunun rahatlattığını düşünüyor. Bunu desteklemek için, bunların etkisi sadece az sayıda araştırmayla kanıtlanmış olsa da bitkisel preparatlar, örneğin kekik bazlı preparatlar alınabilir. Önemli olan aşırıya kaçmadan yeterli miktarda sıvı almaktır. Vücudun korunması da iyileşmeye yardımcı olur. Öksürük sırasında sigara içmekten (aktif ya da pasif) kaçınılmalıdır. Bakteriyel enfeksiyonlarda genellikle can kurtaran antibiyotiklerin solunum yolları enfeksiyonlarına neden olan virüslere karşı bir etkisi yoktur. Bu nedenle öksürük halinde normalde antibiyotik tedavisine gerek yoktur. Bu noktada, solunum yollarındaki tüm enfeksiyonların sadece ufak bir oranını oluşturan bakteriyel akciğer enfeksiyonu istisnadır.

Charité (Berlin) ve Friedrich-Schiller üniversitesi (Jena), bu hasta bilgilendirme belgesinden RAI projesi çerçevesinde sorumludur (www.rai-projekt.de). Telif hakkı Lindgrün GmbH Berlin 2016.
Bu hasta bilgilendirme belgesi yetişkin bir kişi olarak size özeldir. Doktor desteği olmadan kendi kendine teşhis veya tedavi için kullanılmamalıdır. Sorularınız varsa lütfen tedavinizi yapan doktorla görüşün. Buradaki öneriler ve bilgiler çocuklar için önem arz etmeye bilir.

9 / 2016

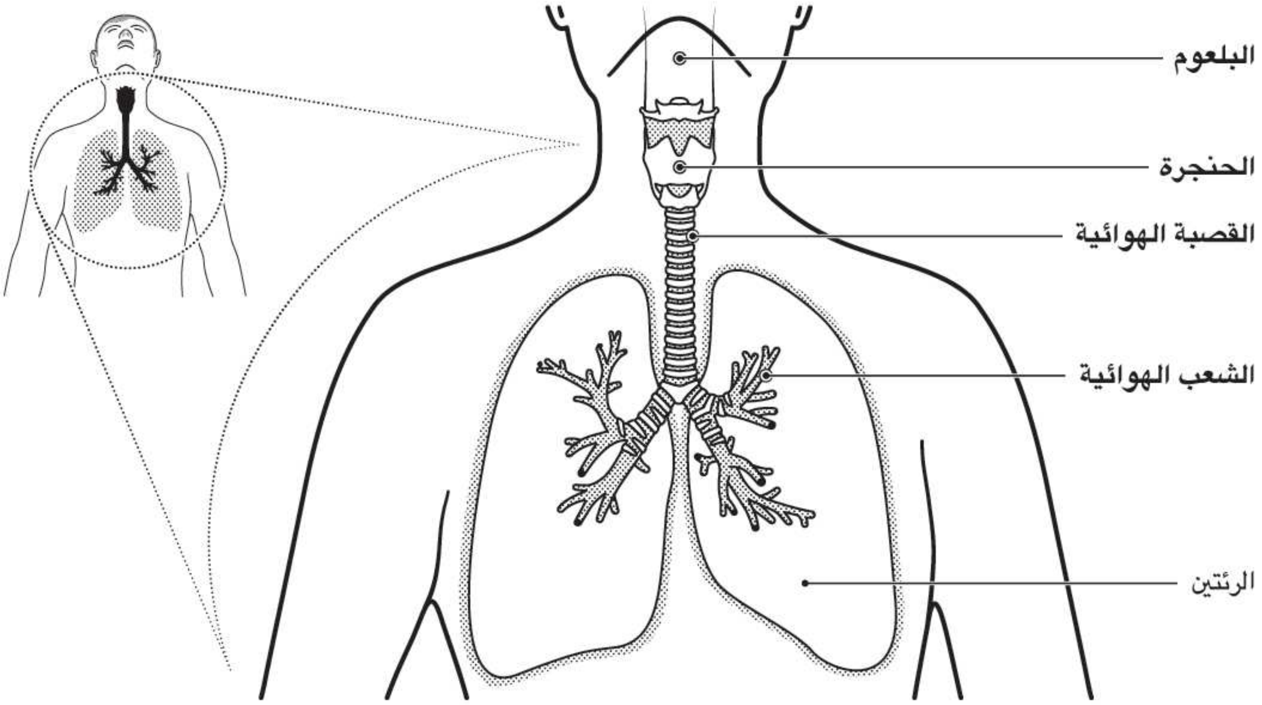

السعال - ما مسبباته؟

يعد السعال ردة فعل احترازية هامة يقوم بها جسم الإنسان، فمن أجل تطهير المسارات الهوائية من رذاذ المخاط والأتربة والأجسام الغريبة يقوم الجسد باطلاق هواء انفجاري بسرعة الإعصار، وتعد المستقبلات المتواجدة بالشعب الهوائية والقصبة الهوائة والحنجرة هي المسئولة عن تلك المهمة، حيث يتم استثارتها عن طريق وجود تجمع مخاطي على سبيل المثال، فترسل إشارة للمخ ينتج عنها اطلاق السعال. وبجانب السعال اللإرادي يوجد أيضا سعال متعمد ويفعله الإنسان بإرادته.

كيف يصاب الإنسان بالسعال؟

لا يعد السعال مرضا في حد ذاته، بل هو عارض لأمراض مختلفة، ومن ضمن هذه الأمراض عدوى المسارات الهوائية كنزلات البرد والتهاب الشعب الهوائية، ففي الغالب تسبب العدوى الفيروسية التهابات في المسارات الهوائية مما يؤثر في الحال أو بعد وقت قصير بالزيادة على إنتاج الإفرازات المخاطية. ويعد كلا من زيادة الإفرازات والالتهابات محفزا رئيسا للسعال، حيث تقوم الأهداب المتواجدة بأعداد مليونية في الغشاء المخاطي بمهمة طهارة ونقاء الشعب الهوائية، وحين تصاب تلك المنطقة بالعدوى فإن ذلك الجهاز التطهيري يتعطل، وحينئذ يشكل السعال حلا لتلك المشكلة، حيث يعمل على تطهير المسالك الهوائية من ذلك الرذاذ المخاطي والبكتيريا والفيروسات المصاحبة له.

ما أعراض السعال وكيف يظهر؟

غالبا ما يكون السعال المصاحب لعدوى المسارات الهوائية جافًا في بادئ الأمر، وبعد مرور فترة قصيرة يزداد إنتاجية المخاط (البلغم) بشكل ملحوظ، والذي يصل إلى الفم بفعل اندفاعات السعال القوية. أحيانا يظهر المخاط باللون الأبيض ويمكن أيضا أن يظهر بألوان مختلفة كالأصفر والأخضر، ولا يعني تغير اللون أن العدوى بكتيرية.

يصاحب السعال أعراض أخرى للعدوى كالزكام والتهاب الحلق وبحة الصوت والصداع والضعف العام وأحيانا الحمى، وغالبا ما يشفى المرء من تلك الأعراض في غضون أيام، إلا أن السعال قد يستمر لمدة قد تصل إلى أربعة أسابيع بعد الإصابة بنزلات البرد أو الإنفلونزا أو التهاب الشعب الهوائية.

السعال - ماذا علي أن أفعل؟

يشفى الإنسان في العادة من أمراض المسارات الهوائية والسعال المصاحب لها دون أدوية، وغالبا ما تكون بلا عواقب، ولأن السعال في حقيقة الأمر ردة فعل هامة للجسد من أجل تطهير المسارات الهوائية، فلابد إذا من اقتصار تناول مضادات السعال على الحالات الاستثنائية، وليس المريض في حاجة غالبا إلى استخدام أدوية الغرغرة المضادة للبلغم، إلا أن الكثير من المرضى يجدون في المعالجة باستنشاق البخار وسيلة جيدة ومريحة. ومما يساعد المريض في حالة الشكوى من السعال تناول المركبات النباتية مثل المركبات المستخدم فيها الزعتر، وذلك رغم قلة الدراسات العلمية حول فاعلية تلك المركبات. ومن الأهمية بمكان تناول السوائل بشكل كافي وعدم الإفراط فيها، وكذلك راحة الجسد، فإنها مؤثرة بشكل إيجابي في عملية التعافي. أما التدخين - السلبي أو الإيجابي- فيجب تحنبه في حالة السعال. وتعد المضادات الحيوية في حالات العدوى البكتيرية فعالة ومفيدة غالبا، أما في حالة العدوى الفيروسية فلا فائدة فيها، ولهذا فليس ثمة حاجة في تناول المضادات الحيوية في حالة الشكوى من السعال العادي، أما في حالة التهاب الرئة البكتيري، والذي يمثل جزء ضئيلا من عدوى المسارات الهوائية، فينصح باستخدام تلك المضادات.

تتحمل مستشفى برلين وجامعة فريدرش-شيلر (Jena) كافة المسئولية عن المعلومات الطبية المقدمة في هذا المنشور، وذلك في إطار مشروع (RAI) وموقعه ا لكتروني (www.rai-projekt.de). حقوق النشر محفوظة لشركة ليندجرين برلين 2016، شركة ذات مسئولية محدودة. هذه المعلومات الطبية مقدمة لكم باعتباركم كبار بالغين. ولا ينصح باستخدامها في التشخيص الذاتي أو العلاج دون استشارة الطبيب المعالج. هذه النصائح والتوجيهات قد تكون غير مناسبة للأطفال.
