# Supplementary material for: Evaluation of a multimodal intervention to promote rational antibiotic use in primary care
Source: Antimicrob Resist Infect Control. 2021 Apr 6;10:66. doi: 10.1186/s13756-021-00908-9 (PMC8025382; doi:10.1186/s13756-021-00908-9)
Supplement: Supplementary file 2 — Additional file 2. List of questionnaire items (translated from German). [file 13756_2021_908_MOESM2_ESM.docx]

| **Table XX.** List of questionnaire items (translated from German) | | |
| --- | --- | --- |
| **Topic** | **Question** | **Answer categories**^1^ |
| **Sociodemographic data** | **Q1**: Gender | Male / Female |
|  | **Q2**: Year of birth | Number |
|  | **Q3**: Working as a GP since | In years |
|  | **Q4**: Type of practice | Single Practice / Joint Practice / Medical Care Center |
|  | **Q5**: Which specialist training did you complete? | General Practitioner (GP) / Internal Medicine / Doctor in GP Specialist Training / Other |
|  | **Q6:** What is your federal state? | Berlin / Brandenburg / Thuringia |
|  | **Q7**: What is the number of inhabitants in your region of work? | <5,000 / 5,000-19,000 / 20,000-99,000 / 100,000-1 Mil. / > 1 Mil. |
|  | **Q8**: How many visits do you have per quarter? | <400 / 400-800 / 801-1,200 / 1,201-1,600 / >1,600 |
| **Antibiotic resistance** | **Q9**: How relevant is the subject of antibiotic resistance to your daily work? | Highly / Moderately / Little / Not at all |
|  | **Q10**: Do you think your antibiotic prescribing behaviour influences the antibiotic resistance status in your region? | Yes / No / Do not know |
|  | **Q11a:** Do you discuss the subject of AMR with your patients suffering from infections?  … when prescribing an antibiotic: | Very often / Often / Partly / Rarely / Never |
|  | **Q11b:** Do you discuss the subject of AMR with your patients suffering from infections?  … when NOT prescribing an antibiotic: | Very often / Often / Partly / Rarely / Never |
|  | **Q12:** Do you use the strategy of delayed antibiotic prescribing? | Strategy not known / Very often / Often / Sometimes / Rarely / Never |
| **Digital information**  **prescription** | **Q13:** How often did you use digital information prescription? | Daily / Weekly / Monthly / Never, Because: [Text] |
|  | **Q14:** Did the digital information prescription support your communication with the patient? | Yes / No / Do not know |
|  | **Q15:** Was the translation of the digital information prescription (English, Arabic, and Turkish) useful? | Yes / No |
|  | **Q16:** Did you miss a certain language? | Yes / No  If yes, which language: |
|  | **Q17:** Praise and criticism for the digital information prescription: | [Text] |
| **Printed information**  **prescription** | **Q18:** How often did you use printed information prescription? | Daily / Weekly / Monthly / Never, because: [Text] |
|  | **Q19:** Did the printed information prescription support your communication with the patient? | Yes / No / Do not know |
|  | **Q20:** Praise and criticism for the printed information prescription: | [Text] |
| **Self-monitoring App** | **Q21:** How frequently did you use the self-monitoring app for the documentation of antibiotic prescriptions? | Daily / Weekly / Monthly / Never, because: [Text] |
|  | **Q22:** Praise and criticism for the self-monitoring app: | [Text] |
| **Conclusion** | **Q23:** What is your overall impression of the tools (digital and printed information prescription, waiting room patient leaflet, self-monitoring app, posters, GP training on rational antibiotic use)? | Not used / 6-Point scale |
|  | **Q24:** How do you judge the influence of the tools on your antibiotic prescribing behaviour (information prescription, waiting room patient leaflet, self-monitoring app, GP training rational antibiotic use)? | Very strong / Strong / Little / No / Do not know |
|  | **Q25:** What else do you want to tell us? | [Text] |

Single selection, unless otherwise specified
